# From Innate to Adaptive Immune Response in Muscular Dystrophies and Skeletal Muscle Regeneration: The Role of Lymphocytes

**DOI:** 10.1155/2014/438675

**Published:** 2014-06-16

**Authors:** Luca Madaro, Marina Bouché

**Affiliations:** ^1^IRCCS Fondazione Santa Lucia, Via del Fosso di Fiorano 64, 00143 Rome, Italy; ^2^DAHFMO, Unit of Histology and Medical Embryology, Sapienza University of Rome, Via Antonio Scarpa 14, 00161 Rome, Italy

## Abstract

Skeletal muscle is able to restore contractile functionality after injury thanks to its ability to regenerate. Following muscle necrosis, debris is removed by macrophages, and muscle satellite cells (MuSCs), the muscle stem cells, are activated and subsequently proliferate, migrate, and form muscle fibers restoring muscle functionality. In most muscle dystrophies (MDs), MuSCs fail to properly proliferate, differentiate, or replenish the stem cell compartment, leading to fibrotic deposition. However, besides MuSCs, interstitial nonmyogenic cells and inflammatory cells also play a key role in orchestrating muscle repair. A complete understanding of the complexity of these mechanisms should allow the design of interventions to attenuate MDs pathology without disrupting regenerative processes. In this review we will focus on the contribution of immune cells in the onset and progression of MDs, with particular emphasis on Duchenne muscular dystrophy (DMD). We will briefly summarize the current knowledge and recent advances made in our understanding of the involvement of different innate immune cells in MDs and will move on to critically evaluate the possible role of cell populations within the acquired immune response. Revisiting previous observations in the light of recent evidence will likely change our current view of the onset and progression of the disease.

## 1. Background

Only a small number of immune cells reside within intact skeletal muscle, but they are recruited during injury and play important roles in the regeneration process, critically contributing to its resolution. Upon injury, immune cells rapidly infiltrate the muscle to remove necrotic tissue and secrete soluble factors that serve initially to activate muscle satellite cells (MuSCs) [1–3]. As such, immune cells constitute a transient local environment for MuSCs. Satellite cells and immune cells attract one another through chemokines (chemoattraction). Satellite cells have been demonstrated to secrete a panel of proinflammatory cytokines, such as IL-1, IL-6, and TNF-*α* to facilitate immune cell infiltration and function [4, 5]. In turn, immune cells secrete a wealth of diffusible factors, such as growth factors, IL-6, globular adiponectin, extracellular matrix (ECM) components, and ECM remodeling MMPs. These diffusible factors generate ECM chemoattractive fragments, which help satellite cells escape from the basal lamina of myofibers, and promote satellite cell proliferation [6]. In addition, cell-to-cell contact between immune and satellite cells protects satellite cells from apoptosis [7]. All these events must be timely regulated and alterations in quality, amount, and time lead to impaired regeneration, increased muscle wasting, and deposition of fibrotic tissue, as it occurs in muscle aging or in muscular dystrophies, such as Duchenne muscular dystrophy (DMD) [8, 9]. DMD is a lethal X-linked genetic disorder caused by deficiency of dystrophin, a critical component of the dystrophin glycoprotein complex (DGC), acting as a link between the cytoskeleton and extracellular matrix in skeletal and cardiac muscles [10]. A consequence of the DGC inefficiency is muscle fragility, contraction-induced damage, necrosis, and inflammation. Although satellite cells compensate for muscle fiber loss in the early stages of disease, eventually these progenitors become exhausted [11]. Moreover, aberrant intracellular signalling cascades that regulate both inflammatory and immune processes contribute substantially to the degenerative process. As a result, fibrous and fatty connective tissue overtakes the functional myofibers. These changes culminate in progressive muscle wasting, with the majority of patients being wheelchair-bound in their early teens, succumbing to cardiac/respiratory failure in their twenties [12]. Among the animal models of DMD, the* mdx* mouse model is the best characterized. It lacks dystrophin expression, and, though with a milder phenotype, exhibits extensive limb muscle degeneration and inflammation, as well as myocardial lesions [13–15]. Available data sets, although limited and not comprehensive, suggest that early immune cell infiltration in DMD patients and* mdx* mice represent an important, but underappreciated, aspect of dystrophic muscle pathology. In fact, although lack of dystrophin makes myofibers susceptible to fragility and degeneration when contracting, this mechanical defect hypothesis for dystrophic muscle death has been unable to explain many aspects of the pathophysiology of DMD and emerging clues attribute an active role to the immune response in these events [16]. From a therapeutic point of view, a clear understanding of the cell populations and of the mechanisms involved in the inflammatory response in DMD may allow the design of valuable anti-inflammatory therapeutic strategies to ameliorate muscular dystrophy. Although blunting inflammation would not restore the primary defect, the emerging clue is that multiple strategies, addressing different aspects of the pathology, which may eventually converge, may be successful.

## 2. Innate Immune Response in Muscular Dystrophies

As a consequence of the dystrophin-null myofibers fragility, DMD muscle is characterized by continuous cycles of myofibers necrosis and repair. Myofibers undergoing degeneration/necrosis, independently of the injury insult, release Th1 inflammatory stimuli, which recruit neutrophils and monocytes/macrophages required to clear cell debris, followed by a Th2 immune response which promotes muscle healing [17]. Much of the current information about the role of immune cell populations of the innate response in muscle repair comes from studies on healthy regenerating muscle [1, 18–20]. Although many similarities were found between acute and chronic muscle injury, the kinetics, quality, and outcome diversify in many aspects [21, 22].

### 2.1. Neutrophils

In acute injury, neutrophils, identified as Ly6C+/F4/80- cells, are the first cells to invade injured muscle, followed by macrophages. In acutely injured muscle in mice, they begin to appear at elevated numbers within 2 h of muscle damage, typically peaking in concentration between 6 and 24 h after injury and then rapidly decline in numbers [23]. Their function mostly involves phagocytic activity to remove debris but also release of TNF*α*, as a Th1 stimuli, and production of myeloperoxidase (MPO), inducing muscle membrane damage and increasing macrophage proinflammatory activity [24–26]. As in acute injury, neutrophils, together with macrophages, invade* mdx* dystrophic muscle as early as 2 weeks of age [24]. Indeed, initial muscle injury and membrane lysis are caused by superoxide production mediated by these early infiltrating neutrophils. Previous studies demonstrated that anti-GR1 antibody-mediated depletion of neutrophils, starting at age 19 days, significantly reduces muscle necrosis at age ~21 days and subsequent regeneration at age ~28 days [25]. However, GR-1 is highly expressed in neutrophils, but it is also expressed in macrophages [27, 28], so whether the observed phenotype was specifically related to neutrophils depletion is not as clear. In any case, although neutrophils activity appears to overlap with that of M1 macrophages (see below), since they are one of the earliest infiltrating cell types, they might promote initial muscle lesion, playing an important role in the early onset of the pathology.

### 2.2. Macrophages

A comprehensive survey of the current knowledge on macrophage involvement in MDs and muscle regeneration is beyond the scope of this review. The reader is referred to many systematic reviews already available on this topic [16, 21, 29]. Briefly, two subpopulations of macrophages have been identified in regenerating muscle tissue that may influence muscle degeneration and regeneration depending on the proportion of these cells present [18, 30]. As a simplistic point of view, the M1 population are proinflammatory, characterized by the expression of iNOS and secretion of proinflammatory cytokines (e.g., TNF*α*, IL-1*β*, and IL-6), and promote muscle cell lysis; by contrast, the M2 population is characterized by the expression of arginase-1, CD163, and CD206 mannose receptor (usually in noninflammatory, repair conditions) and/or anti-inflammatory cytokines (e.g., IL-10) [19, 21, 31, 32]. They are believed to enhance muscle regeneration, by inducing satellite cell proliferation [19]. However, beyond this broad definition, mainly determined from* in vitro* polarization experiments, macrophages exhibit a wide variety of intermediate phenotype, including M2b and M2c, M1 and M2 being the extremes of a continuum in activation states. M2b macrophages are known to release large amounts of IL-10, which promotes the proliferation of nonmyeloid cells, although, like M1 macrophages, they can also release proinflammatory cytokines, such as IL-1*β* and TNF*α*. IL-10 can also induce M2c macrophages, which have anti-inflammatory functions [33]. However, it has been recently highlighted that several markers should be examined at the cellular level in order to properly assess the macrophage inflammatory state* in vivo*, since the population remains heterogeneous [34]. While in acute muscle injury the sequential waves of M1, followed by M2 macrophage invasion, leads to resolution of inflammation and efficient muscle repair, in muscular dystrophies, repetitive cycles of myofiber degeneration lead to muscle invasion by M1 macrophages together with M2a macrophages, which may reduce the cytotoxic activity of M1 macrophages [35]. Therefore, the inflammatory* milieu* in dystrophic muscle is similar, but not the same as found in acute injury. Subsequent invasion of dystrophic muscle by M2c macrophages is associated with progression to the regenerative phase in pathophysiology. However, the number of M2 macrophages declines upon resolution of the damage in acute-injured muscle, while in* mdx* muscle their number increases with age, and promotes fibrosis [36]. Thus, increased and persistent presence of macrophages modifies the intensity, duration, and interactions of the different released factors, leading to increased myofibers necrosis, ECM accumulation, and replacement of muscle with fibrotic and fat tissue. Early studies showed that depletion of macrophages from* mdx* mice before the onset of histopathology caused great reduction in muscle pathology in 4-week-old mice [37], reinforcing the notion that macrophages induce muscle lesions, along with clearing debris. However, given that myofiber degeneration occurs in the absence of dystrophin, depletion of macrophages might not be as beneficial in long term, as it would prevent removal of debris. Indeed, whether or not phagocytosis is required for proper muscle repair is still debatable. Depletion of phagocytes was shown to slow cellular debris removal following freeze-injury of muscle but to have no effect on early postinjury regeneration, in terms of satellite cells activation [38]. By contrast, other studies have concluded that reducing the numbers of phagocytic leukocytes (neutrophils and monocytes/macrophages) in mice prior to toxin-induced muscle injury slows the removal of cellular debris and slows muscle regeneration, since reduced frequency of central-nucleated muscle fibers was observed [18, 39]. However, considering that, as mentioned before, phagocytic leukocytes also promote muscle damage, it could be argued that reduced number of regenerating fibers was dependent on reduced muscle damage because of the depletion of leukocytes. In this context, a recent study by Mounier et al. showed that inhibiting phagocytic activity in macrophages prevents macrophage skewing from a pro- to anti-inflammatory phenotype at the time of resolution of inflammation, in regenerating healthy muscle, suggesting that phagocytosis is required not only to clear debris, but also to promote resolution of the damage [34].

Although these studies are related to healthy regenerating muscle, deciphering the mechanisms regulating macrophages activation and plasticity should greatly facilitate the design of novel therapeutic strategies to modify the dystrophic muscle environment, reduce muscle damage, and increase muscle repair.

### 2.3. Mast Cells

Very little is known about mast cell involvement in muscular dystrophies. Nevertheless, mast cell proliferation and degranulation are often observed in areas of grouped fiber necrosis [40]. Mast cell degranulation involves the release of histamine and cytokines (including TNF*α*), which contribute to a pro-inflammatory environment that promotes muscle necrosis. In addition, proteases released during mast cell degranulation induce membrane lysis of nearby cells promoting local areas of ischemia [41]. Indeed, treatment of* mdx* mice prior to disease onset (age 19 days) with sodium cromoglycate (cromolyn), a blocker of mast cell degranulation, results in ~59% decrease in cumulative TA muscle damage by age 28 days [42]. Further studies are needed to gain insight into the mechanisms that regulate the microenvironment and ischemic conditions that result in fiber necrosis in muscular dystrophies. In a mouse model of arthritis, an inflammatory disease not related to muscle, mast cell-mediated increase of vascular permeability has been shown to be involved in the recruitment of inflammatory cells into the site of inflammation [43]. Interestingly, it has been recently shown that mast cells deficiency in a mouse model of polymyositis results in reduced susceptibility to C-protein-induced myositis, as compared with WT [44]. The reduced susceptibility to C-protein-induced myositis was associated with a reduced number of macrophages and CD8+ T cells, mainly due to reduced vascular permeability. The observed reduced susceptibility was restored by reconstitution of mast cells, highlighting the crucial role of these cells in promoting massive inflammatory cells infiltration [44]. Polymyositis (PM) belongs to the class of idiopathic inflammatory myopathies, IIM, which also includes adult and juvenile dermatomyositis (DM), myositis, and inclusion body myositis (IBM), a heterogeneous group of acquired disorders characterized by chronic inflammation of striated muscle leading to predominantly proximal muscle weakness. Although the precise pathogenesis is unknown, many evidences support their autoimmune basis and they likely result from immune-mediated processes initiated by environmental factors in genetically susceptible individuals [45, 46].

Increase in vascular permeability is certainly one of the events contributing to the immune cell infiltration in dystrophic muscle, as well. These aspects have been poorly addressed in muscular dystrophy, as only recently the disease was recognized as an “inflammatory” disease; investigating the disease from this new perspective may give unexpected insights into the mechanisms regulating its onset and progression.

### 2.4. Eosinophils

Eosinophils are generally associated with immune responses to infection by parasites or with allergic reactions [47]. However, they can play a significant role in the cellular immune response, in which their involvement appears to be tightly coupled both temporally and functionally to T lymphocyte activity; indeed, they are activated by the Th2 cytokine IL-5 [48, 49]. Although eosinophilia occurs only rarely and inexplicably in muscle disease, eosinophil invasion was found in both DMD and* mdx* dystrophies [50, 51]. Previous studies observed that eosinophils increased within* mdx* dystrophic muscle at about 4 weeks of age, together with cytotoxic T cells invasion, and, although their number decreases during the regenerative phase, their concentration remains higher at 30–32 weeks of age, as compared to healthy muscle of age-matched wild type mice, depending on the muscle examined [51]. Interestingly, transplantation of splenocytes derived from* mdx* mice into irradiated WT animal induced an increased number of eosinophils in muscle, as compared to irradiated control mice, suggesting that eosinophils invasion is dependent on lymphocytes activity [51]. Indeed, prednisone treatment (the major drug used in DMD patients) reduces eosinophil infiltration [52]. Moreover, despite the original expectation that eosinophilia in dystrophic muscle was a nonspecific consequence of the Th2 inflammatory environment, eosinophils were shown to modulate injury and regeneration. In particular, genetic ablation of major basic protein-1 (MBP-1), a cytolytic protein expressed by eosinophils, caused an increase in the numbers of cytotoxic T cells in dystrophic muscle [50]. That finding is significant to* mdx* dystrophy because a Th1-driven cellular immune response in which cytotoxic T cells promote apoptosis of* mdx* muscle fibers is an early feature of the disease that is attenuated as muscle regeneration begins. Thus, eosinophils may mediate the regeneration of dystrophic muscle by promoting the transition from Th1 to Th2 inflammatory environment. An interesting insight on the possible role of eosinophils in promoting muscle regeneration was recently suggested by Heredia and colleagues [17]. They showed that eosinophils invade acute injured muscle very early, even before neutrophils invasion. This observation is in contrast to other studies which showed, using the same injury model, that eosinophils are exceeding scarce in muscle following toxin injection and they appear to increase after other inflammatory cell populations [34]. However, using the eosinophil-deficient ΔdblGATA1 mouse model, which presents a 90% reduction in eosinophils, Heredia et al. observed persistent and unresolved muscle damage after acute injury. The authors identified IL-4 as the key cytokine produced by eosinophils, whose lack is known to prevent muscle regeneration [53]. Surprisingly, they demonstrate that the primary target of this cytokine produced by eosinophils at the very early stage of muscle damage is not macrophage, as expected, but fibroadipogenic precursor (FAP) cells. In fact, while lack of IL-4 receptor in macrophages did not result in regenerative defect, the lack of this receptor in FAPs is deleterious and leads to fat deposition by promoting their differentiation into adipocytes. FAPs are recently identified multipotent mesenchymal cells residing in skeletal muscle interstitium, who represent a critical component of the cellular niche required for effective satellite cell-mediated regeneration [54–57]. Thus, eosinophils may form a transitional niche for proliferating FAPs via secretion of IL-4; in the presence of IL-4, FAPs proliferate as fibroblasts to support myogenesis [17]. So far, whether these observations are relevant to muscular dystrophy is a matter of speculation; it can be hypothesized that, in dystrophic muscle, the eosinophils promote the transition from a Th1 to a Th2 inflammatory environment, which would in turn promote FAPs proliferation as fibroblasts to support myogenesis. On the other hand, persistence of eosinophils within the dystrophic muscle would sustain fibroblast proliferation, which promotes fibrotic tissue deposition, accelerating the clinical decline of the disease [36]. Thus, further studies are needed to gain insight into the role of eosinophils in the pathophysiology of muscular dystrophy and to verify whether manipulating eosinophils activity would be therapeutically beneficial.

## 3. Lymphocytes and the Adaptive Immune Response in Muscle Repair

### 3.1. T-Cell Response in DMD

It is widely believed that lymphocytes do not play a relevant role in healthy regenerating muscle, due to the inability of skeletal muscle to activate a T-cell response. Neither MHC class I nor class II molecules has been detected on muscle fibers from healthy muscle tissues [58, 59]. By contrast, appearance of MHC class I and/or II was observed in muscle tissue of patients with idiopathic inflammatory myopathies (IIM), where an autoimmune pathogenesis is now recognized, but also in regenerating fibers of patients with DMD [58]. These findings, together with the observation that an inflammatory* milieu* may upregulate HLA-DR and costimulatory molecules (ICOS-L and CD40), led to the idea that myoblasts can become facultative APCs in both MHCI and II-dependent immune reactions, forming functional immunological synapses with T cells [59, 60]. Indeed, T cells are found in degenerating muscle after acute injury, but their recruitment is more robust and persistent in chronic diseases, such as myositis or muscular dystrophies, where also B cells can be detected [61, 62].

The recruitment of T cells into injured muscle implies an adaptive immune response, which normally depends on antigen exposure. While this mechanism appears now conceivable in inflammatory myositis, it is less clear in regenerating muscle after acute injury or in chronic diseases such as muscular dystrophies. Many observations suggest that muscle-specific autoantigens drive T-cell expansion in IIM [46], but no such observations are available in regenerating muscle or in muscular dystrophies yet. Furthermore, whether or not T-cell infiltrate critically contributes to muscle damage in muscular dystrophies is still debatable. Persistence of T cells in dystrophic muscle may actually modulate inflammatory* milieu* and immune cells activity but may also directly interfere with muscle cell function through lymphocyte-released cytokines and chemokines [63, 64]. Indeed, it was recently shown that conditioned medium from activated T-cells, previously cultured in anti-CD3 coated dishes in the presence of IL2 [65], induced proliferation of C2C12 muscle cell, a mouse derived muscle cell line widely used as a model of myogenesis* in vitro*, and prevents myogenic differentiation, as compared to conditioned medium derived from resting T cells [66].

Very early studies correlated the reduction in T cells observed in prednisone-treated DMD patients, with reduction in muscle necrosis and fibrosis [67, 68]. Further studies identified T cells in muscles of several DMD patients, characterized by a specific T-cell receptor (TCR) rearrangement [69]. The overrepresentation of a T-cell population expressing a restricted set of TCR variable genes might indicate a selective T-cell response directed to a muscle-specific antigen. Their persistence in DMD muscle could derive from either clonal expansion or conserved antigen recognition, or from the emergence of a regulatory population. Interestingly, specific TCR arrangement, different from the one found in DMD, was initially found in the T cells invading muscle in PM patients [70]. Since then, many studies aimed to characterize T-cell populations and their role in MDs and muscle regeneration, though the results were not as exhaustive and were sometimes contradictory.

### 3.2. T-Cell Response in* mdx* Muscle

Elevated concentrations of activated cytotoxic CD8+ and helper CD4+ T cells (Th) are present in affected muscles of* mdx* mice aged 4–8 weeks but rapidly decrease in concentration by 14 weeks of age [63]. However, no increase in these cells has been observed in axillary or inguinal lymph nodes of* mdx* mice, suggesting that their activation is occurring in muscle tissue, and not systemically [63]. CD8+ T cells are the first to invade dystrophic muscle, peaking at 4 weeks of age; their activation is generally driven by a Th1 cellular immune response to kill their target cells through perforin-mediated processes [51]. Around 2 weeks later, CD4+ T cells also invade dystrophic muscle; T helper CD4+ T cells can generally differentiate into Th effector inflammatory cells, mainly Th1 and Th2, or into regulatory T cells (Treg), both of which participate in immune responses. Th1 cells are known to support macrophage M1 polarization by producing IL-1, IL-2, TNF-*α*, and INF-*γ*, while Th2 produce IL-4, IL-13, and IL-6 sustaining the M2 macrophage polarization [71]. On the other hand, Treg cells are required for the resolution of the immune response. These cells are characterized by the expression of Foxp3 transcription factor and produce anti-inflammatory cytokines such as IL-10 [72]. They were originally described as regulators of T-cell activity but were later recognized to also regulate B cells and several other innate immune response players [73].

One of the first studies addressing the possible role of lymphocytes in* mdx* showed that antibody-mediated depletion of CD8+ or CD4+ cells in* mdx* mice, beginning at 6 days of age and continuing until the age of 4 weeks, resulted in a 75% and 61% reduction in muscle histopathology, respectively [63]. This positive outcome suggested an important role for these cells in the development of muscle lesions. In another study,* scid/mdx* mice, which are deficient in functional T and B lymphocytes, were shown to develop much less diaphragm fibrosis at 1 year of age and a decrease in activated TGF*β* in skeletal muscle, compared with* mdx* mice. Improvement in muscle regeneration was also observed in these mice, but not in muscle functionality [74]. Accordingly, in* nu/nu/mdx* mice, the lack of functional T cells alone was associated with less diaphragm fibrosis at 3 months of age [75]. Altogether, these results support the pathogenic role of T cells in* mdx* muscle and reveal this lymphocyte subclass as an important source of TGF*β*1. By contrast, more recently, an additional dystrophic immunodeficient mouse model was generated, the Rag2(−)Il2rb(−)Dmd(−) mouse, which lacks T, B, and NK cells, and also carries a mutant Dmd allele that prevents the production of any dystrophin isoform [76]. Although not systemically analyzed, the authors reported that there was no difference in the pathological features of the disease in these mice, compared to* mdx* mice. Indeed, previous studies have shown that thymectomy at 1 month of age, which completely depletes circulating T cells, failed to prevent diaphragm fibrosis at 6 months of age [75]. Although this result suggested that the fibrotic process is self-sustaining from a very early stage, it can be argued that depleting circulating T cells at 1 month of age was already late, as early muscle invading T cells may eventually expand within the muscle and contribute to fibrous tissue deposition. However, the possibility of muscle-specific T cells clonal expansion has been poorly investigated, so far.

T cells represent approximately 3% of all infiltrating cells in* mdx* muscle, with over half present as double-negative T cells (lacking both CD4 and CD8 expression), 8%–10% of which were recently identified as being NKT-like cells, which express both T and NK markers [77]. Interestingly, a predominant T-cell population expressing V*β*8.1/8.2 was identified within the muscle infiltrate in* mdx* mice at 1 month of age. These V*β*8.1/8.2+ cells are not generally overrepresented in circulating cells, and, therefore, it is likely that these cells are either selectively home to or expand within* mdx* muscle. These cells express high level of osteopontin (OPN), which modulates cellular immune profiles in* mdx* muscles; in fact, its ablation in* mdx* mice results in significant reduction in neutrophils, CD3+/V*β*8.1/8.2+ cells, and NKT-like cells, but not macrophages. Interestingly, an increase in CD3+/CD4+/FoxP3+ Treg cells was observed, highlighting the importance of modulating T cells subclasses in muscular dystrophy. In this context, OPN appears to be an immune modulator that specifically impacts the concentration of NKT-like cells, neutrophils, and Tregs in dystrophic muscle [77]. As a result, lack of OPN in* mdx* mice reduces intramuscular TGF*β* and fibrosis in both muscle and heart at 6 months of age, and importantly, it improves muscle strength. V*β*8.1/8.2+ cells are not the only source of OPN in dystrophic muscle as other immune cells, as well as muscle tissue, which could also produce it. Thus, besides the identification of OPN as a potential therapeutic target to ameliorate inflammation and progression of muscular dystrophy, these data clearly demonstrated a crucial role of T-cell subclasses in modulating the inflammatory* milieu* in dystrophic muscle. In line with this view, treatment with the immunosuppressant drug Rapamycin ameliorates the* mdx* phenotype with a reduction of both CD4+ and CD8+ cells, but not of regulatory Foxp3+ cells [64]. In this context, we recently showed that lack of protein kinase C *θ* (PKC*θ*) in* mdx* mice prevents muscle degeneration and inflammation, thus improving muscle regeneration and, importantly, muscle performance [78]. PKC*θ* is a member of the PKC family, predominantly expressed in muscle, where we and others have shown that it is an upstream regulator of several intracellular pathways leading to muscle homeostasis [78–83]. Furthermore, PKC *θ*, which is a key regulator of T-cell activation and proliferation, is being proposed as an attractive target to prevent immune response and alloreactivity [84, 85]. Indeed, it appears to be required for the development of a robust inflammatory response in vivo [86, 87]. Interestingly, PKC*θ* ^−/−^ mice fail to develop experimental allergic encephalomyelitis, display drastically reduced lung inflammation after induction of allergic asthma, and have a significantly diminished response in experimental colitis, and a type II collagen induced arthritis model [86, 88, 89]. Of note, PKC*θ* ^−/−^ mice can still mount a normal protective immune response to clear viral infections, and, importantly, maintain Treg function, since PKC*θ* is known to inhibit Treg differentiation [90, 91]. Indeed, we showed by bone marrow transplantation experiments that the improved phenotype observed in the double mutant mdx/PKC*θ* ^−/−^mice is primarily due to lack of PKC*θ* in the hematopoietic cell compartment [78]. Thus, the possibility that lack of PKC*θ* in T cells may alter the T-cell subclasses within dystrophic muscle, and, in turn, the inflammatory* milieu*, including macrophage subclasses and function, is currently under investigation.

### 3.3. T-Cell Response in Dysferlinopathies

The involvement of lymphocytes activity appears to be a general feature in muscular dystrophies. In fact, it has been recently demonstrated that lack of functional T/B cells improves regeneration in a mouse model of dysferlinopathies [92]. These diseases belong to the so-called limb girdle muscular dystrophies (LGMDs), characterized by predominant weakness and wasting of muscles of the pelvic and shoulder girdle. Among them, LGMD-2B and Miyoshi myopathy (MM) develop due to defects in the dysferlin gene, coding for a membrane protein mainly required for vesicle traffic and membrane repair [93]. Inflammatory cells were detected in both MM and LGMD patients, scattered or organized into clusters, around necrotic fibers. Inflammatory infiltrates around vessels mainly consisted of macrophages, whereas CD4+ and CD8+ cells were found in endomysial infiltrates. Abnormal MHC-I expression was observed in degenerating/regenerating fibers usually close to inflammatory cluster cells but was absent in normal fibers [58, 59]. Indeed, the dysferlin-deficient AJ mice, one of the mouse models of dysferlinopathies, lacking functional T/B cells (AJ/SCID mice) develop a milder pathologic phenotype with increased muscle regeneration and reduced percentage of proinflammatory M1 macrophages. Importantly, these mice showed an increase in muscle force. The rescue of the phenotype was primarily due to a reduced IL-6 production by macrophages [74]. Indeed, liposome-clodronate macrophage depletion impairs IL-6 production also in control AJ mice, supporting the notion that the beneficial effect of T/B cells absence is due to a switch in macrophage phenotype and function.

### 3.4. The Emerging Role of Treg Cells in the Immune Response to Muscle Injury

As mentioned above, although it has long been recognized that lymphocytes invade acutely injured muscle, very little is known about their role in these conditions, mainly due to the very small number of these cells within the muscle, making their characterization difficult.

Recently, several mouse models have been generated to further understand the mechanisms underlying T cells recruitment, expansion, and persistence in acutely injured muscle.

Interestingly, it was recently shown that acute myofiber damage itself transiently activates muscle-antigen-specific CD8^+^ T cells in draining lymph nodes, supporting the idea of a transient autoreactive immune response upon muscle injury [94]. These activated CD8^+^ T cells transiently invade acute injured muscle, but their negative impact on muscle repair is promptly controlled by immunosuppressive cues [94]. The authors proposed that inadequate control of this CD8^+^ T-cell response might favor the emergence of sustained autoimmune myositis. In line with this view, Young et al. showed that aberrant muscle antigen exposure is sufficient to induce myositis in a Treg deficient* milieu* [95]. They generated a FoxP3/Syt VII double mutant, in which lack of synaptotagmin VII (Syt VII, a member of synaptotagmin family of membrane-trafficking proteins) impairs membrane resealing thus exposing endogenous muscle antigens [96]. Interestingly, while Syt VII mutant mice develop a mild, self-limiting inflammatory response involving muscle, FoxP3/Syt VII double mutants develop a significant inflammatory response in muscle, histologically resembling polymyositis in humans. Taken together, these studies suggest that, although muscle injury may evoke an autoreactive immune response to endogenous muscle antigens, this reaction is blunted within muscle through local immune-suppressive Treg dependent mechanism. In support of this, a muscle specific Treg population capable of potentiating muscle repair was recently identified [72]. Following cardiotoxin (CTX) muscle injury, a Treg cell population began to accumulate at day 4 after injury, just as the myeloid cell infiltrate switched from a proinflammatory to a proregenerative phenotype. Their frequency peaked at 2 weeks after injury, accounting for about 50% of the CD4^+^ T cells. However, while the number of CD4 T cells dropped to levels characteristic of uninjured muscle by 28 days after CTX injection, the number of Treg cells remained elevated. Interestingly, the increase in their number was not due to further recruitment, rather to clonal expansion within the muscle. Although these muscle Treg cells expressed the canonical gene signature characteristic of Treg cells, microarray gene expression analysis showed that the muscle Treg cells were closely related to Treg cells derived from visceral adipose tissue (VAT), but different from Treg cells in lymphoid organs. The authors provide a large data set of genes differentially expressed in VAT, muscle, colonic lamina propria, and prediabetic NOD pancreas derived Treg cells, supporting the notion that the Treg compartment is heterogeneous, has multiple functions, and exerts effects beyond the boundaries of the immune system [97]. Importantly, using the Foxp3-DTR mouse, in which Diphtheria Toxin is expressed under the control of the promoter of the Treg-specific Foxp3 gene, they showed that ablation of Treg cells by Diphtheria Toxin injection, after muscle injury, prevented muscle regeneration [72]. These authors identified a key molecule released by muscle Treg, responsible for the observed beneficial effect, named Amphiregulin (Areg), an EGF family growth factor. Administration of Areg in mice lacking Tregs restored efficient muscle regeneration, suggesting that this growth factor is part of fundamental cues during muscle repair. In this context, previous studies have suggested that Areg might act directly on muscle cells [98, 99]. Interestingly, a Treg population was enriched in dystrophic muscle derived from dysferlin-deficient and* mdx* mice. In particular, in* mdx* muscle, during both the acute and the chronic phase of the disease, this enriched Treg appears to be similar to muscle Treg. Although their number is very low, loss- and gain-of-function experiments showed that reducing Treg cells exacerbates the disease, while enriching them ameliorates it, as measured by serum creatine kinase level, an enzyme released into the blood upon muscle damage and a standard indicator of muscle damage in dystrophic mouse models, as well as by gene expression analysis. Taken together, these findings raise the possibility that a muscle antigen might be involved in recruiting Treg cells to the site of injury and/or retaining them therein. Since endogenous muscle antigens are released in physiological conditions upon injury as well as during pathological conditions by mechanical stress, such as in muscular dystrophies, it is conceivable that Treg cells, which are recruited and/or clonally expanded in muscle following injury, function to prevent a potential systemic autoimmune response by suppressing and regulating the inflammatory response. Taken together, these studies are very important and identify Treg cells and their products as potential new players in the orchestrated series of events underlying muscle repair in both acute and chronic context.

## 4. Concluding Remarks

Tissue damage induces a series of complex events including inflammation, which culminate in deposition of ECM. If this process is faulty, excessive and persistent ECM deposition takes place, and normal tissue is substituted by collagen scar, resulting in tissue dysfunction. In muscle, dysregulated repair with persistent fibrosis rather than efficient regeneration plays a prominent role in the clinical decline and reduced life expectancy associated with severe muscular dystrophies. A deep knowledge of the complex events following muscle damage will hopefully allow to design strategies aimed at promoting efficient regeneration.

Although inflammation is now considered a pathological feature of muscle repair, the role and regulation of this process have not been sufficiently examined. Moreover, the relative role of the innate or adaptive immunity in muscle regeneration and dystrophies is still unclear. Although blunting inflammation would not be a “cure” for these diseases, the emerging picture is that multiple strategies, addressing different aspects of the repair process, which may eventually converge, may be successful. In this context, although macrophages are emerging as indispensable for damage control and tissue remodelling following muscle injury, and as principal mediators of pathological skeletal remodelling in diseases such as IIMs and dystrophies, the involvement of other immune cells in promoting or preventing muscle damage resolution is also emerging.

In the last two decades, much research has pointed out the active interactions between muscle cells and the immune system and has clarified some of the mechanisms involved in IIMs. According to most of these studies, muscle cells are now emerging as possible facultative APCs, able, within an inflammatory* milieu,* to drive the activation and proliferation of CD4+ T cells, previously primed against exogenous or endogenous peptides [100]. Whether similar mechanisms may take place in muscle regeneration and muscular dystrophies is not clear yet. It is well known that T cells invade muscle upon both acute injury and chronic diseases. T cells can be recruited by muscle or other infiltrating cell populations, through cytokine release, but their persistence and activity might be sustained by an antigen-specific response. The possibility that an adaptive response to endogenous muscle antigens released upon muscle fibers degeneration, which may represent a common feature in acute injury as well as in IIM or MDs, would attribute to all of these conditions an auto-reactive component. In acute injury, the efficient repair process and the ability of MuSCs to proliferate and differentiate may be sustained by local Treg dependent immune regulation. In fact, autoreactive T cells are normally negatively selected in the thymus; if they escape to the periphery, they are normally eliminated by induction of apoptosis or rendered nonfunctional by the induction of anergy [101]. By contrast, in IIM, muscle fibers are direct targets of adaptive response, and the persistence and clonal expansion of autoreactive T cells prevent muscle healing. Although speculative at this stage, it may be hypothesised that in MDs, the continuous cycles of muscle fiber degeneration, due to muscle fiber fragility, allow for a prolonged and sustained endogenous muscle-specific antigen exposure, leading to the persistence and clonal expansion of potential muscle-antigen reactive T cells, eventually leading to breakdown in peripheral tolerance to self-antigens. Thus, if cytotoxic T cells escape the mechanisms of peripheral tolerance, a cellular immune response will accompany the innate immune response to tissue damage. The presence of alloreactive cytotoxic T cells in* mdx* muscle [102], the ability to transfer pathology from* mdx* mice to healthy mice by adoptive transfer of immune cells primed with muscle homogenates [63], and the presence of a well-conserved peptide in the hypervariable domain of the T-cell receptor of cytotoxic T cells from DMD patients [69] all support the possibility that a breakdown of peripheral tolerance occurs in muscular dystrophy. Furthermore, the observation that muscle specific clonal expansion of Treg cell population occurs during muscle regeneration and in MDs also suggests that a specific adaptive immune response is operating during muscle repair.

**Figure 1:**
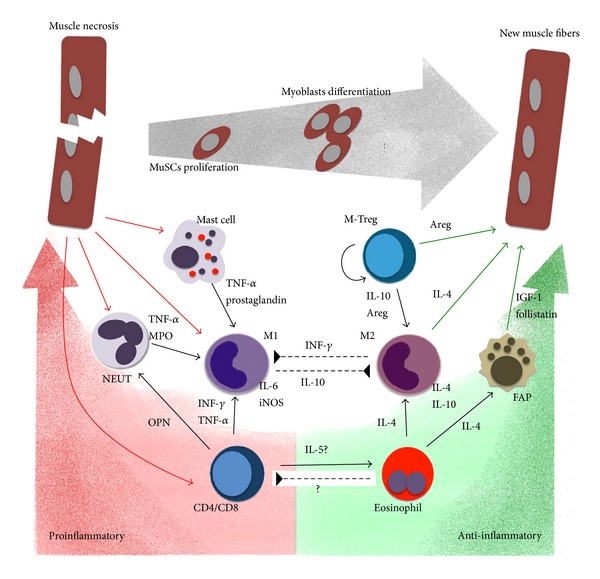
The complexity of the inflammatory milieu in muscle repair. During the initial phases of muscle damage, degenerating fibers release chemokines and cytokines recruiting mast cells, neutrophils (NEUT), and CD8/CD4 T cells which sustain proinflammatory M1 phenotype of recruited monocytes, thus promoting muscle fibers necrosis and debris clearing. These events are primarily mediated by IL-6, NO, TNF*α*, and IL-1*β* release. On the other hand, eosinophils are also recruited, which induce FAPs to sustain MuSCs proliferation via IL-4 release. Eosinophils-induced FAPs-produced follistatin promotes MuSCs differentiation, together with the clonally expanding mTreg which produce Areg. Areg also sustains MuSC proliferation and maintains/induces M2 anti-inflammatory/prohealing phenotype, supporting resolution of inflammation. Unbalance in the kinetics, quality, and activity of any of these actors would prevent resolution of inflammation, making muscle environment unfavourable for muscle regeneration, as it occurs in muscular dystrophies.

The discovery of the possible autoantigens that may evoke an adaptive immune response in MDs will facilitate therapeutic intervention. Similarly, the dissection of the T-cell response, which contributes to the complexity of the inflammatory* milieu* (outlined in Figure 1), will also allow the design of novel therapeutic strategies that would modulate the immune response to create a more favorable environment for MuSCs to differentiate and efficiently regenerate muscle lesions.
